# Resources that Help Sustain Environmental Volunteer Activist Leaders

**DOI:** 10.1007/s11266-023-00561-3

**Published:** 2023-03-01

**Authors:** Robyn E. Gulliver, Charlie Pittaway, Kelly S. Fielding, Winnifred R. Louis

**Affiliations:** 1 School of Communication and Arts, The University of Queensland, St. Lucia, Brisbane, QLD 4072 Australia; 2 School of Psychology, The University of Queensland, St. Lucia, Brisbane, QLD 4072 Australia

**Keywords:** Environmental collective action, Resource mobilization theory, Volunteer leadership, Social movements, Thematic analysis

## Abstract

Environmental activism organizations depend on recruiting and retaining individuals willing to engage in leadership tasks on a voluntary basis. This study examined the resources which help or hinder sustained environmental volunteer activist leadership behaviors. Interviews with 21 environmental volunteer activist leaders were analyzed within a Resource Mobilization Theory framework. While six resources supporting sustained engagement in volunteer activist leadership behaviors were identified, only three were sought by all participants: time, community support, and social relationships. Money, volunteers and network connections were considered valuable resources, however their acquisition generated significant additional administrative burdens. Social relationships sustained volunteer activist leaders through fostering feelings of positive emotions connected with the group. We conclude with suggestions for organizations seeking to increase retention of activist volunteer leaders: namely larger organizations sharing their resources to reduce administrative demands on volunteer activist leaders in smaller organizations; developing movement infrastructure groups to build and sustain networks; and the prioritization of positive relationships within volunteer teams.

## Introduction

Volunteer activist leaders are critical for sustaining and growing social movements (Carman & Nesbit, 2013; Louis et al., 2020; Mati et al., 2016). The environmental movement in particular has grown substantially over the last decade while having a heavy reliance of volunteer groups (Gulliver et al., 2021b). As such, volunteer leaders of environmental activist groups in particular play a critical role in sustaining environmental mobilization over time (Ganz & McKenna, 2018; Gulliver et al., 2020; Maloney & Rossteutscher, 2007). However, activist leaders who engage in intense, long-term activism often experience fatigue and burnout (Chen & Gorski, 2015; Saunders et al., 2012; see also Vestergren et al., 2017 for a review). Indeed, research shows that new not-for-profit organizations which depend on volunteers often struggle to persist over time (Bennett, 2016). Effectively supporting volunteers engaging in leadership tasks for these groups remains a significant challenge. Yet the substantial literature investigating paid leadership is in contrast to the comparative lack of research on volunteer activist leaders, who operate in a context with different motivations and incentives (Mati et al., 2016; Nesbit et al., 2016; Smith, 2015). Previous researchers have pointed to the unique dimensions of voluntary activist leadership (Chiariello, 2008; Ganz & McKenna, 2018; Nesbit et al., 2016). Volunteer activist leaders must recruit other volunteers, mentor them, and retain them (Bunnage, 2014). They help form and maintain relations with partners while maintaining a group’s vision and shaping norms about collective action, all over the long term without renumeration (Nesbit et al., 2016; Reicher et al., 2005; Thomas & Louis, 2013).

New research is needed to provide insight into what influences volunteer activist leaders to persist with and balance the myriad tasks required for ongoing social movement mobilization (Nesbit et al., 2016; Posner, 2015). The current study aims to address this research gap by investigating the factors that support volunteer activist leaders in continuing their efforts. To do this, interviews with environmental volunteer activist leaders were conducted and analyzed through the lens of Resource Mobilization Theory (RMT: McCarthy & Zald, 1977). RMT argues that resources underpin the emergence and survival of social movement organizations. While RMT has commonly been applied to consider organizations as a whole, given that volunteer leaders are crucial for acquiring and converting resources into mobilization and action (Edwards & McCarthy, 2004a; Gulliver et al., 2020; Morris & Staggenborg, 2004), we reasoned that insights from RMT may also identify factors that support individuals to persist as volunteer activist leaders, and provide insights into how to overcome barriers to sustaining such leadership.

This study contributes to the field in two important ways. First, the broader leadership literature indicates that leadership behaviors are motivated by factors such as status, high renumeration, and agency (Haslam et al., 2010). However, in the volunteer space, the research primarily examines the motivations and characteristics of leaders who are either paid to manage volunteers, or lead non-activist volunteer organizations, such as community, hobby, or sports groups (e.g., De Clerck et al., 2021). These voluntary ‘associations’ may have well-defined roles and leadership positions, such as ‘President’ and ‘Secretary’ (Nesbit, 2017; Nesbit et al., 2016). In contrast, volunteer activist leaders have fuzzy, ill-defined roles, no renumeration, no particular status, and a record of ongoing struggle and failure (Gulliver et al., 2022; Louis, 2009; Nesbit et al., 2016). Furthermore, research examining volunteer participation in activism (also called ‘activist/protest volunteering’, see Mati et al., 2016) most commonly focuses on what motivates individuals to participate in collective action, usually on a one-off, or occasional, basis (e.g., Van Zomeren, 2013). These occasional volunteer behaviors may be driven by different motivations than sustained volunteer activist leadership behaviors (Alisat & Riemer, 2015; Gulliver et al., 2022). The current research therefore explores a type of leadership that has not been the focus of past research.

Our second contribution to the field is to extend the RMT framework to interrogate the complex dynamics of volunteer activist leadership, the motivations of individuals who are simultaneously resource managers and mobilization resources themselves. Given the theoretical validity of RMT in explaining long-term mobilization, this approach will advance our understanding of how RMT applies to both volunteer activist organizations and the individuals who form them. This understanding can then assist volunteer activist organizations in prioritizing the acquisition of resources which may have the greatest impact in sustaining their volunteer leaders.

## Theoretical Background and Research Questions

### Resource Mobilization Theory

RMT examines the influence of resources on the emergence and activities of social movement organizations (Edwards & McCarthy, 2004a; McCarthy & Zald, 2001). While earlier social movement theories suggested mobilization was driven by irrational, alienated individuals sharing a common grievance, RMT argued that grievances alone could not explain mobilization; instead, mobilization is a rational process of organization which develops only when aggrieved individuals can acquire sufficient outside resources to act. Organizations with access to fewer resources and less capacity to manage them are less able to act on their grievances.

In general, a ‘resource’ refers to any asset able to be drawn upon by a person or organization to aid their functioning (Macmillan, 2022; see also, Cress & Snow, 1996). Three broad resource categories were identified in early RMT literature: human resources such as volunteers and staff, material resources such as money or land, and organizational resources such as network connections between groups (McCarthy & Zald, 1987). Additional categories such as cultural or moral resources have also been identified (Bomberg & Hague, 2018; Edwards & McCarthy, 2004a), which can include symbolic objects such as images and icons (Edwards & Kane, 2014). Cultural resources can also include social-psychological factors such as spiritual values (Bomberg & Hague, 2018), leaders’ charisma (Ahmed, 2022), a group’s vision and mission (Edwards & McCarthy, 2004a), and shared identities (Louis et al., 2016).

As well as aiding mobilization, RMT postulates that organizations with greater access to resources will be more likely to persist (McCarthy & Zald, 1987). Indeed, some scholars argue that the primary barrier to ongoing activism is lack of resources (Almog-Bar & Schmid, 2014; Bass et al., 2007). RMT therefore may provide a useful lens to consider the factors influencing ongoing volunteer activist leadership, given its central role in maintaining activist organizations over time.

### Resources and Volunteer Activist Leadership

In this section we consider what resources have been identified as important to sustaining activist groups and reflect on the extent to which they may apply to individuals who engage in leadership tasks for those groups. Sourcing human resources—individuals willing to engage in both one-off and ongoing activities on behalf of the group—is central for social movement organizations given their aim to mobilize large numbers of individuals to act collectively for a cause (Balduck et al., 2015; Brown et al., 2016).

In general, RMT research on the use of resources in volunteer contexts has often assumed that paid human resources (staff) will be responsible for the acquisition and management of financial, material, and other resources that attract volunteers and structure volunteers’ experiences (Nesbit et al., 2016). In contrast, social movement organizations primarily depend on volunteers to acquire and manage these resources themselves (Gulliver et al., 2020). Social movement organizations are often also heavily dependent on long-term volunteers for organizational tasks (Bräuer, 2008). Indeed, some studies show that over two-thirds of nonprofit groups have no paid staff at all, while the majority also have no formal organization status that enables them to acquire or manage financial resources (Gulliver et al., 2020, 2021a; Maloney & Rossteutscher, 2007). Accordingly, research has suggested that the most common responsibility of volunteer activist leaders is the acquisition and management of human resources; namely, recruiting and retaining other volunteers (Nesbit et al., 2016).

However, it cannot be assumed that additional volunteers are necessarily a positive resource for volunteer activist leaders. Volunteer teams often have high turnover rates (Christens & Speer, 2011; Han, 2017), and while some volunteers have useful skills, others can assist only in unspecialized tasks (Jenkins, 1983). Furthermore, volunteer leaders must manage volunteers in an environment where many traditional management processes are less effective (Nesbit et al., 2016). They may not have well-defined authority, particularly in decentralized or non-hierarchical activist groups. As a result of these factors, some scholars have also argued that specific leadership related resources such as charismatic authority may play an important role in mobilizing individuals in the social movement context (Ahmed, 2022; Andreas, 2007).

In groups using radical tactics, leaders may face a higher degree of personal risk when organizing volunteers (Smith, 2015). In fact, traditional ‘command and control’ management styles may be highly ineffective in volunteer activist groups. The growth of movements describing themselves as decentralized, or even ‘leaderless’ (Fotaki & Foroughi, 2021; Gulliver et al., 2021b) suggests that some groups may even reject the idea of leaders entirely. Instead, volunteer activist leaders may find that volunteer demands reduce their capacity to offer long-term leadership.

Similarly, material resources such as funding and office space may be challenging for volunteer activist leaders to manage without paid staff to assist. While increased financial resources have been shown to increase a group’s ability to mobilize individuals (e.g., Kohl-Arenas, 2014), research from the broader social movement and activism literature suggests that it can also have the opposite effect, consuming time and energy in fund-raising, reporting, and accounting requirements, while influencing the type of advocacy organizations can engage in (Child & Grønbjerg, 2007; Mati, 2012). Critically, research demonstrating that more financial resources are linked to increased activism is based on organizations with paid staff to manage these resources (Bass et al., 2007; Child & Grønbjerg, 2007).

Another common resource identified in RMT literature is network connections, referring to connections between groups within a larger movement. These connections act as a resource as they can increase a group’s recruitment opportunities and facilitate information-sharing (Edwards & McCarthy, 2004b; Seyfang & Longhurst, 2013; Wakefield et al., 2006). However, as with human and financial resources, volunteer activist leaders may find network connections difficult to manage. Lack of time to establish and maintain connections alongside competition among groups can impact network strength (Mihaylov & Perkins, 2015). Given these mixed findings, the extent to which network connections and material resources enable volunteer activist leaders to sustain their efforts in the long-term, remains unknown.

Outside of RMT research, some studies suggest that positive interpersonal connections can influence individuals’ participation in activism over time. Feelings of empowerment, solidarity (Drury et al., 2005), commitment, connection to the cause (Driscoll, 2018), belonging, and friendship with others in the group (Aiken & Taylor, 2019; Schussman & Soule, 2005) can be drawn upon by volunteer activist leaders as resources which facilitate their ongoing participation. Collective identities and shared struggles may also play important roles as psychological resources binding individuals to movements (Carroll & Hackett, 2006; Van Zomeren, 2013). These findings suggest that volunteer activist leaders with stronger and more positive social relationships with group members may continue their volunteer efforts for longer.

### The Current Study

A wide range of resources have been identified as important for sustaining groups engaging in activism. Yet despite volunteer leaders’ critical roles in organizing these groups, we know comparatively little about the resources which specifically support and sustain them. Given that sustaining activism over time is difficult (Han et al., 2017), this study seeks to advance our understanding of resources which may assist environmental activist groups to retain their voluntary leaders (Posner, 2015). This is important when considering that leaders may exit groups due to mental or emotional exhaustion (Scherer et al., 2016). The loss of these leaders can then lead to reduced organizational effectiveness (Han et al., 2011), or even the decline and disappearance of volunteer organizations in their entirety (Bennett, 2016; Smith, 2015). Better understanding what resources volunteer activist leaders need to continue their leadership is therefore beneficial in light of the importance of social mobilization in driving the changes needed to address our environmental crisis (Ripple et al., 2017).

In this study we focus specifically on environmental activist volunteer leaders’ experiences. We conducted a qualitative analysis of semi-structured interviews with 21 environmental volunteer activist leaders to identify what resources they use, and how these resources influence their sustained voluntary engagement in leadership behaviors. To help shed light on this topic, we examine the following three research questions:

- What resources do volunteer activist leaders identify as important to their role?
- What barriers and opportunities do volunteer activist leaders associate with these resources?
- How do they perceive that these resources influence their volunteer leadership activities and ongoing service?

## Method

### Participants

Volunteer activist leaders (*N* = 21) were recruited through contacting specific environmental groups identified in the Australian environmental movement (Gulliver et al., 2019, 2020). To ensure a diversity of participants we contacted groups who were active in different Australian regions and focused on different environmental issues. Groups were asked to share the interview request with their teams to ascertain interest, with 21 activists offering to participate. All interviewees engaged in leadership activities such as organizing or leading projects or the group. The sample comprised 14 women and 7 men, aged 23 to 80. Their experience of activism with local grassroots groups entirely dependent on volunteers ranged from 5 to 40 years. A total of nine participants undertook activism on conservation and protection issues, such as the preservation of forests and marine environments. Seven participants focused on climate change, with the remaining five participants focusing separately on sustainability, mining, nuclear, pollution and renewable energy issues.

### Procedure

Interview questions covered five broad areas: the activists’ personal motivation to engage in activism, their campaigning goals, their strategic and tactical campaign approaches, their engagement with other groups within the broader environmental movement, and their experiences as volunteer leaders. Questions were asked in broad terms in order to gather a wide perspective on participants use of resources without specifically leading activists to prioritize or identify any one resource over another. The interviews were semi-structured, enabling participants to freely discuss their perceptions, experiences, and opinions about environmental activism. Within each of the five broad areas participants were prompted with follow up inquiries, such as ‘could you explain that further?’, or ‘why do you think the outcome was a success/failure?’ All interviews were conducted by the first author and took place in May 2018 either in person or via telephone. The interviews lasted on average one hour and were recorded and transcribed.

### Analytic Procedure

Transcribed interview data was coded through a thematic qualitative analysis process following Braun and colleagues (Braun & Clarke, 2020; Braun et al., 2014). This process involved a first stage of repeated close reading of all interviews to gain a comprehensive sense of the material. A list of resources (‘themes’) that interviewees said they drew upon in order to maintain or enhance their leadership activities was then collated. Following this, a coding frame based on the themes identified in round one was created to guide a second round of deductive coding undertaken using NVivo software (O’Connor & Joffe, 2020). Text excerpts identifying resources mentioned by participants were then reviewed to identify whether participants stated that the resource was useful or not useful in supporting their ongoing activities. All coding was undertaken by the first author of this study, with 20% of the data second-coded in three stages of review and discussion. Intercoder reliability was selected as the method to ensure credibility of results and prompt discussion of a common conceptual framework across the research team (O’Connor & Joffe, 2020). Assessing reliability was considered relevant for this study, given that RMT literature provides well-defined resources relevant for organizations that our data could be compared against. The final Krippendorff’s Alpha and Cohen’s Kappa reliability scores was 0.848, indicating a high level of agreement between coders (Hayes & Krippendorff, 2007).

## Results

### Resources

Six resources important for helping volunteer activist leaders achieve success emerged from the data: time, network connections, money, volunteers, social relationships, and community support. Time was the only resource unanimously described by all 21 participants as both sought after and providing a universally positive influence on their ability to engage in volunteer leadership tasks; however, time was also the resource all volunteer leaders described as being the most difficult to acquire. Participants highlighted how managing the resources of network connections, money, and volunteers was a difficult balance to maintain within the limited time resources available, and in some cases reduced their ability to prioritize mobilization. In the following sections we highlight each of these six resources and the ambivalence felt relating to their acquisition and management.

### Time

Time was the resource most desired by volunteer activist leaders, with all participants saying they would prioritize gaining additional time over other resources. Furthermore, unlike the two other resources mentioned by all participants (network connections and money) there was no ambivalence or management challenges related to increased acquisition of time. Unsurprisingly, a lack of time not only affected activists’ ability to recruit, manage and retain their group members, but also to design and implement their campaigns.

“We didn't take that step [of creating the campaign] because of the time constraints. I think that's the problem a lot of volunteers have on top of everything else that they're doing. Just literally the fight that's involved, and the emotional stress and strain and everything else. It's pretty demoralizing.” (VL7)

### Network Connections

The support provided by network connections with other groups was also mentioned by all 21 participants; however, significant ambivalence was expressed regarding the benefits and constraints of this resource. Many participants highlighted the positive support they have received from other environmental groups and the impact this made on their ability to succeed:

“We do quite a bit with the [other environmental group]. They were great, they actually mentored us. We got a grant which gave us funding to set our group up. Through that we engaged the [other environmental group], to send up one of their members to mentor us for 3 or 4 days to get some planning going” (VL3)

Yet despite the benefits of this resource, most participants also noted that collaborating with other groups was challenging and often ineffective. Collaboration was particularly fraught when volunteer activist leaders sought to engage with staffed environmental groups with greater resources. Maintaining effective network connections took substantial time, especially when these connections broke down, or conflicts arose. In addition, some participants argued that poor network connections in general had resulted in the failure to build a strong, united and powerful environmental campaigning sector.

“About 10 years ago there was an alliance of all the big groups set up to speak to the government. But for some reason that fell over or didn't achieve anything… I don't know how to do it, but I know we're not doing it. I don't know what the answer is. The small groups are pivotally important in their area, they're doing fabulous things. It’s just there’s no big picture.” (VL5)

### Money

All 21 volunteer activist leaders discussed this resource. As with network connections, participants were ambivalent about prioritizing the acquisition of money, with many participants also noting the complexity of how money can influence tactics and movement legitimacy. Some participants listed concrete campaign wins and other positive outcomes that money had enabled.

“We managed to get a $20,000 grant to run a research project in the [local forest] with the [partner organization]. We set up 30 research sites and involved over 100 community members. Those sites are still there and still being researched over 10 years later” (VL9)

However, participants also noted how acquiring, managing, and acquitting funds often drained their time and energy which could be better focused on other activities. The influence of money on tactical choice and organizational security was also identified by some participants. Environmental groups found that their access to funds affected their autonomy and strategic choice. Some volunteer activist leaders highlighted that while money enabled the acquisition of material resources such as office space and advertising, the absence of funding provided greater freedom in their mobilization choices and personal values.

“The sort of work we do is direct action… a donor would have to be quite secretive about it and certainly nobody would give you tax deduction… I don’t think people who are trying to bring down a really deeply unfair and ecocidal economic system are typically paid very well!” (VL4)

### Volunteers

Twenty participants mentioned volunteer resources. Volunteers were critical in helping volunteer activist leaders achieve their goals; however, volunteer recruitment and retention were consistently identified as challenging. This was predominantly due to the high demands that were placed on volunteer activist leaders to mentor, train, and manage new volunteers.

Participants distinguished between volunteers who were able to complete well-defined tasks and volunteers who had initiative and were able to work autonomously. Participants argued that volunteers generally required high levels of training, support, and management, and so volunteers with initiative were highly valued.

“We make it clear [to new volunteers] that we’re already at full capacity ourselves. If you want to be part of this, you need to become part of the project team and take responsibility for yourself. We’ll skill you up and train you, but we’re not going to be doing a long mentoring process. We need you just to jump in, boots and all … Because that is quite a big ask, our volunteer base for the actual projects, committee, and the management is quite small.” (VL3)

Volunteers’ skills were also identified as important resources which could increase how efficiently volunteer activist leaders could organize their groups’ activities. For example, skills related to financial management and income generation were highly valued:

“We do everything without money, except with [this campaign]. [Our volunteer is] such a genius, he decided that we needed to do a crowd funding thing. And we did, and we got $10,000. So that campaign was very professional.” (VL15)

However, many participants highlighted how voluntary leadership skills were different from task-focused volunteering. Voluntary leadership skills were highly sought after by other volunteer leaders, primarily because they had firsthand experience of the volume of work leadership roles entailed.

“It's easy to be a volunteer but being that ‘next level volunteer’ is harder because you have more mental work. You can't necessarily work as much in your other job, because you've got to spend your time replying to emails, thinking about how the meetings are going to be run, contacting people for this and that, organizing events, thinking about how it's going to go and matching up all the dots. That sort of thing is a huge amount of work, huge.” (VL1)

### Social Relationships

While RMT notes the importance of network connections between groups, these connections also exist between leaders and their group members as well as between individuals within groups. Participants frequently noted that the quality of these peer connections was of critical importance in their own ongoing engagement in activist leadership. Twenty participants specifically highlighted the importance of positive social relationships in sustaining their activities, primarily relationships with other volunteers within their groups. These relationships generated ongoing positive emotions, and participants described these relationships as critical to maintaining their ongoing desire to continue their volunteer work. Relationships functioned as personal rewards accrued through their volunteering.

“I just love it, I just love it. I've learnt so much, I spend time with really great people. I hang out with scientists, I am in the forest, I can read the forest more. I just love it. I can't believe I got so lucky.” (VL5)

Participants highlighted a range of positive emotions connected with these social relationships. These included emotions such as joy, solidarity, and love, which some participants linked to their sense of purpose and meaning.

“You know, it's been very enriching to my life also to have such a strong purpose and to work with really awesome people.” (VL3)

### Community Support

Community support was identified by 18 participants as an important and beneficial resource. This was primarily due to the perception that community support could influence an environmental group’s ability to prosper, as well as increase opportunities to obtain other resources such as volunteers:

“I've lived in other communities where I wouldn't be contemplating forming a group … but there have just been some historical events in [our town] that have allowed people to make some interesting decisions about shared ownership, recycling, and environmental care that make our [campaign goal] seem possible.” (VL13)

## Discussion

Volunteer activist leaders form the core of social and environmental movements (Curtin & McGarty, 2016; Saunders et al., 2012) and yet little is known about the factors which influence their sustained engagement in leadership behaviors. In this study we interviewed 21 environmental volunteer activist leaders to identify the resources that helped or hindered their ongoing engagement in leadership activities. Six resources were identified that volunteer leaders identified as important to their role (RQ1). Time, network connections and money were resources mentioned by all participants, while volunteers, social relationships, and community support were mentioned by most, but not all, participants.

Our second research question considered the barriers and opportunities associated with these resources. In line with classical RMT theory (McCarthy & Zald, 1977), financial and human resources were focal for volunteer leaders. Money and volunteers were both seen to offer opportunities for volunteer activist leaders to increase the ability of the group to achieve their aims. However, volunteer activist leaders experienced significant barriers in acquiring and using these resources, most particularly if they did not already have sufficient financial and human resources to assist with administrative burdens. Indeed, unskilled volunteers could consume more resources than they produced, due to training, mentoring and supervision obligations (see also Warburg, 2018).

Our findings demonstrate that resource availability directly influences environmental volunteer activist leaders’ decision making. For example, participants noted how increased financial resources offered opportunities to expand activities while simultaneously presenting barriers to action. Some participants highlighted how financial resources facilitated long-lasting projects (see also, Kohl-Arenas, 2014); however, others noted how financial donors could influence (i.e., prevent) the use of more radical or disruptive mobilization tactics (see also Child & Grønbjerg, 2007; Saunders, 2007). Some volunteer activist leaders sought to avoid this influence by undertaking zero-cost activities or engaging in non-confrontational activities. Others accepted that their choice to engage in radical tactics dictated a reliance on volunteer labor with little financial support. Furthermore, our data provide further evidence of how network connections between and within groups can influence volunteer leadership. Some participants noted how their network connections provided strategic and financial support to their group (see also Edwards & McCarthy, 2004b; Seyfang & Longhurst, 2013; Wakefield et al., 2006). However, they also noted that establishing and retaining network connections demanded significant time, while collaborating with network partners on projects could lead to disagreements around tactics and strategy (e.g., Tranter, 2009).

Research question 3 considered how these resources appeared to affect volunteer leaders’ activities and ongoing service. As highlighted by a number of scholars, choosing between resources involves trade-offs between how the resources could be used, and the administrative burden required to manage them (e.g., Bräuer, 2008). However, three of the six resources—time, positive relationships, and community support—were mentioned as resources which helped volunteer leaders sustain their activities without requiring trade-offs or presenting barriers. While RMT posits that network connections will be beneficial for sustaining groups, our data suggest that the quality of the personal relationships within these networks may also be important. Positive relationships can be a resource of particular value to individuals themselves, and this may be particularly important for individuals in voluntary leadership roles (see also Murdie & Peksen, 2015).

The beneficial role of these resources is consistent with other work in RMT showing relationships—whether between groups or between individuals—are important to volunteer groups (Aikin & Taylor, 2019; Schussman & Soule, 2005). Our research focus was on volunteer leaders; individuals who influence and engage with groups and therefore by necessity need to build relationships with other group members (Haslam et al., 2010; Platow et al., 2015). Accordingly, although our results support RMT in the resources that were identified by volunteer activist leaders, we found that the resource which appeared to play the most important role in sustaining volunteer activist leaders was the personal relationships they formed within their teams. These relationships—more than financial or human resources—generated strong positive emotions which could bind teams together by fostering feelings of joy, love and empowerment.

### Applied Implications

Our findings suggest three areas where organizations can manage resources to better retain their volunteer activist leaders. First, larger organizations with greater resources can play an important role in helping volunteer activist leaders through provision of resources. Of particular value would be providing financial support or skilled volunteers; these two resources may be especially helpful for sustaining smaller volunteer groups at early stages in their development. Our findings demonstrated how balancing financial and human resources created a catch-22 for our participants: resources could only be obtained if volunteer activist leaders already had the resources to acquire them. As a result, organizations with greater resources could seek to assist volunteer leaders by supporting the retention of skilled, independent, and motivated volunteers, particularly skilled volunteers able to shoulder the burden of acquiring and managing financial and material resources to be distributed to others (see also Bräuer, 2008; Maloney & Rossteutscher, 2007). Another avenue for support could be via building partnerships with businesses willing to offer pro bono services to environmental organizations, which could provide financial management services (see also Kohl-Arenas, 2014) as well as help develop networks and build organizational capacity, skills, and knowledge (Seyfang & Longhurst, 2013).

Second, participants noted how network connections—while challenging to initiate and manage—had afforded mentoring and project development support at critical moments in their group’s development. Those aiming to support a growing environmental movement could seek to organize and manage networking services, such as events which bring together volunteer groups without adding substantial demands on volunteer activist leaders. Reducing competition between volunteer groups, for example over grant opportunities (e.g., Mihaylov & Perkins, 2015) could also assist in building network strength. One mechanism for enhancing network connections is through fostering the development of movement infrastructure groups. These can include groups which exclusively focus on the provision of training, human resources management, or funding services. The development of movement infrastructure groups also offers promising avenues for supporting volunteer activist leaders in the post-COVID-19 context. These groups could seek to build shared online spaces that facilitate relationship building and peer-support between volunteer activist leaders working on similar issues in different geographic contexts. Similarly, centralized activism volunteering hubs could be developed, potentially increasing volunteer recruitment by listing both in-person and remote volunteer activism opportunities.

Third, and most importantly, positive social relationships within groups played a critical role in sustaining environmental volunteer activist leaders. Positive social relationships have been shown to support long-term activists to gain a sense of purpose from the environmental activism they engage in (Bunnage, 2014; Driscoll, 2020). These positive feelings may help balance the anger and frustration which can drive initial participation in activism. Relatedly, some research indicates that intrinsic motivation (e.g., Self Determination Theory: Ryan & Deci, 2000) may be an important driver of activist behavior, particularly when combined with enjoyment in undertaking those behaviors (Sheldon et al., 2016). Strong relationships with others in the group may therefore be a prerequisite to building the sense of belonging, purpose and enjoyment which may sustain volunteer environmental activist leaders and their groups. Accordingly, organizations should seek to develop and offer free and easy to access services specifically aimed at nurturing positive relationships in teams. These can include group relationship building strategies, mediation and on demand psychological support. These services could be provided by larger established organizations, which may help smaller groups foster positive group relationships and support any volunteer activist leaders who may seek help.

### Strengths, Limitations and Future Research

The present research benefits from rich interview data with a range of environmental volunteer activist leaders, many with decades of experience. However, given leaders’ close relationships with group members (Haslam et al., 2010; Platow et al., 2015), it would be of value to undertake a comparative analysis which compares insights on the resources which support volunteer activist leaders to those that support activists who are not leaders. Future research should also consider comparative analysis with volunteer activist leaders who have exited or are new to their roles. In addition, our study seeks to examine experienced environmental activist volunteers’ perceptions and experiences of the resources that sustain their leadership behaviors. Future research could build on literature noting the importance of leadership characteristics such as charisma (e.g., Ahmed, 2022) to examine how particular leadership characteristics influence the mobilization and retention of group members over time.

Furthermore, while the focus on committed activists from a range of grassroots and professional groups is a strength of this study, our findings are grounded in a single socio-cultural context. In Australia, environmental activism is largely able to occur freely despite a divisive political atmosphere (Beeson & McDonald, 2013; Tranter, 2011). Activists therefore experience leadership in very different ways from those operating in more authoritarian or violent contexts, where environmental activists and leaders have been kidnapped and murdered in recent years (Global Witness, 2018). Moreover, our study population was ethnically homogenous and predominantly female identifying. Taken together, these limitations imply caution in generalizing the findings reported in this study to a broader population of individuals involved in environmental activism leadership, and a need for cross-cultural and comparative research in future.

## Conclusion

Volunteer activist leaders play a critical role in driving the grassroot environmental activism required to address our global environmental crisis. In this study we examined the resources which help or hinder volunteer activist leadership of environmental groups. While financial, human and network connection resources could support volunteer activist leaders to sustain their activities, they were not always prioritized given the additional administrative requirements they generated. Conversely, increased time, community support and positive relationships were the resources most desired by volunteer activist leaders. Participants highlighted the importance of positive social relationships which generated positive, joyful experiences sustaining their volunteer efforts and promoting their sense of purpose. These findings indicate the importance of resources in understanding why some volunteer activist leaders persist over long periods of time despite ongoing declining environmental indicators and a worsening environmental crisis.
